# Mitochondria-Targeted Antioxidants as Potential Therapy for the Treatment of Traumatic Brain Injury

**DOI:** 10.3390/antiox8050124

**Published:** 2019-05-08

**Authors:** Elena V. Stelmashook, Nickolay K. Isaev, Elisaveta E. Genrikhs, Svetlana V. Novikova

**Affiliations:** 1 Research Center of Neurology,w 125367, Russia; nisaev61@mail.ru (N.K.I.); genrikhs@neurology.ru (E.E.G.); novikova.s.v@neurology.ru (S.V.N.); 2 Biological Faculty, N.A. Belozersky Institute of Physico-Chemical Biology, M.V. Lomonosovw State University,w 119991, Russia

**Keywords:** mitochondria-targeted antioxidant, traumatic brain injury, mitochondria

## Abstract

The aim of this article is to review the publications describing the use of mitochondria-targeted antioxidant therapy after traumatic brain injury (TBI). Recent works demonstrated that mitochondria-targeted antioxidants are very effective in reducing the negative effects associated with the development of secondary damage caused by TBI. Using various animal models of TBI, mitochondria-targeted antioxidants were shown to prevent cardiolipin oxidation in the brain and neuronal death, as well as to markedly reduce behavioral deficits and cortical lesion volume, brain water content, and DNA damage. In the future, not only a more detailed study of the mechanisms of action of various types of such antioxidants needs to be conducted, but also their therapeutic values and toxicological properties are to be determined. Moreover, the optimal therapeutic effect needs to be achieved in the shortest time possible from the onset of damage to the nervous tissue, since secondary brain damage in humans can develop for a long time, days and even months, depending on the severity of the damage.

## 1. Introduction

**Figure 1:**
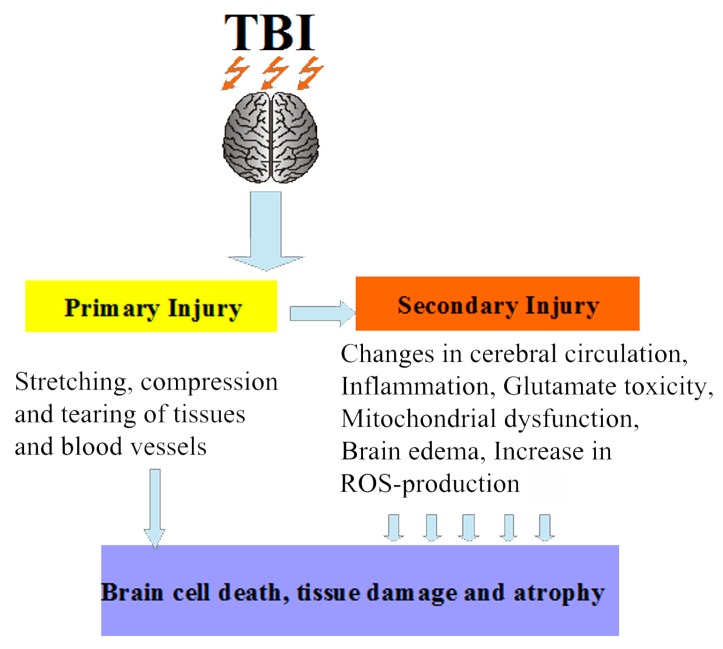
Schematic representation of traumatic brain injury (TBI): the secondary damage involves many complex biochemical and cellular processes that increase the severity of the primary damage. ROS: reactive oxygen species.

It is estimated that from 64 to 79 million people per year suffer from traumatic brain injury (TBI) of varying severity worldwide [1]. The consequences of severe forms of TBI are disability or even death, and the percentage of their occurrence is much higher than for any other traumatic injury. The clinical forms of this pathological state of the brain depend on the severity of the damage and can be represented by a concussion of the brain, contusion of varying severity, compression of the brain caused by various factors, or diffuse axonal injury. It should be noted that TBI increases the risk of diseases of the cardiovascular, respiratory, motor, and digestive systems, as well as of neurological and psychiatric disorders [2]. In addition, according to some data, the likelihood of developing Alzheimer’s-type dementia among people who suffered from TBI increases 2–4 times [3,4]. Therefore, TBI is an important medical and social problem. The cerebral circulatory system is extremely sensitive to damages occurring as a consequence of TBI, so one of the consequences of TBI is the impairment of myogenic constriction of cerebral arteries, which, along with glutamate toxicity, mitochondrial dysfunction, autophagy, brain edema, and inflammation, can be attributed to processes of secondary brain damage [5,6]. In severe TBI, the secondary damage can develop for a long time (days or even months); it leads to brain cell death, tissue damage, and atrophy [7] and involves many complex biochemical and cellular processes that increase the severity of the primary damage (Figure 1). It is known that reactive oxygen species (ROS) can be generated in various cellular compartments, but most of the cellular ROS, approximately 90%, are generated in the mitochondria during the production of ATP through oxidative phosphorylation [8]. The highly reactive radicals can damage mitochondrial macromolecules, including lipids, proteins, and DNA [9]. After TBI, an increase in mitochondrial ROS production is the most important pathogenetic mechanism underlying neurodestruction. Mitochondria are not only powerful generators of ROS in cells, but also targets for these chemically active molecules, since they have a genome unprotected by histones and a high content of cardiolipin, which is an important component of the mitochondrial inner membrane. The increased production of these active molecules following TBI was shown to result in the selective peroxidation of cardiolipin [10], which is involved in the stabilization of complexes of the electron transport chain. Impaired electron transport, in turn, leads to decreased ATP production, increased formation of toxic free radicals, and altered calcium homeostasis. These toxic consequences of the dysfunction of the electron transport chain may sustain further mitochondrial damage, including oxidation of mitochondrial DNA, proteins, and lipids, and opening of the mitochondrial permeability transition pores [11]. The main hazard is the damage to the complexes of the electron transport chain, such as complex I, complex III, and complex IV. If they are damaged or inhibited, the chain cannot work properly, which again leads to the formation of a large number of ROS and, as a result, to neurodegeneration. ROS also activate various molecular signaling pathways associated with cell death [12]. By damaging mitochondria, external ROS can boost the production of free radicals by mitochondria. The way to overcome excess ROS has long been known and involves the use of antioxidants. The clinical efficacy of several antioxidants, such as vitamins C and E, progesterone, N-acetylcysteine, on outcomes of TBI has been evaluated [13]. These studies showed a positive trend in the use of antioxidant therapy, and it was concluded that routine antioxidants can be used as adjuvant therapy in TBI. The usual antioxidants do not have a selectivity of action, and rather high concentrations of these substances are often used to achieve a therapeutic effect. It should be noted that, normally, a limited generation of free radicals is necessary for normal functioning of the cells, and their production is strictly localized in certain compartments. The most important of these compartments are the mitochondria. Mitochondrial redox metabolism, phospholipid metabolism, and proteolytic pathways are found to be the major potential sources of free radicals [14]. Oxidative damage to mitochondria can lead to cell death, and thus mitochondria are an important target for therapeutic intervention in a number of pathologies, including TBI. In this regard, there is a need for the targeted delivery of antioxidants to mitochondria. Such chemical compounds are mitochondria-targeted antioxidants, which can serve as a basis for the creation of a new generation of drugs aimed at the treatment of secondary brain damage caused by TBI.

## 2. Mitochondria-Targeted Antioxidant Therapy in Traumatic Brain Injury

**Figure 2:**
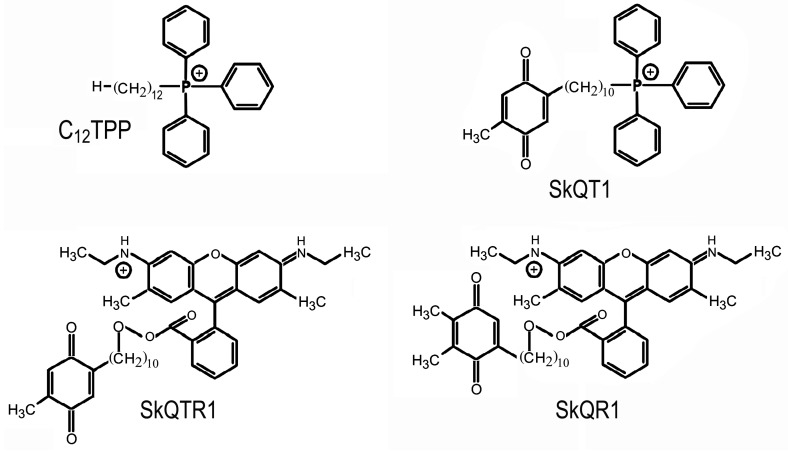
Structure of SkQR1, 10-(6′-plastoquinonyl)decylrhodamine 19; SkQT1, (a mixture of 10-(6′-toluquinonyl)decyltriphenylphosphonium and 10-(5′-toluquinonyl)decyltriphenylphosphonium in the proportion of 1.4:1); SkQTR1, 10-(6′-toluquinonyl)decylrhodamine 19; C12TPP, dodecyltriphenylphosphonium.

At present, a number of compounds that belong to mitochondria-targeted antioxidants have been synthesized. They are able to electrophoretically accumulate in mitochondria; many of them are effective at very low concentrations. The group of mitochondria-targeted antioxidants comprises such substances as SkQs, MitoQ, Mito-Vit-E, SS peptides, and XJB-5-131 [6,15,16,17,18]. The creation of these recently discovered chemical compounds was based on the results of studies with lipophilic phosphonium cations carried out in the 1960s, early 1970s by the group of V.P. Skulachev and E.A. Lieberman. These studies demonstrated that lipophilic ions with a delocalized charge shielded by bulky substituents freely penetrate into mitochondria and submitochondrial particles under the action of the electric field of the inner mitochondrial membrane [19]. Very low concentrations of mitochondria-targeted antioxidants such as MitoQ, SkQ1, SkQR1 were shown to exhibit highly efficient antioxidant activity in aqueous solutions, lipid micelles, liposomes, isolated mitochondria, and cell cultures [20,21,22,23]. The protective effect of these substances was demonstrated in various models of ROS-associated diseases, including the models of such pathological states of the brain as Alzheimer's disease, brain ischemia, TBI [13,15,16,17,18,19,20,21,22,23,24,25,26,27,28,29]. Daily intraperitoneal injections of the mitochondria-targeted antioxidant SkQR1 (100 nmol/kg) for 4 days after a focal trauma of the sensorimotor cortex area were found to improve performance in a test characterizing neurological deficit and to decrease the volume of the damaged cortical area [27]. The antioxidant portion of the SkQR1 molecule is plastoquinone (Figure 2). In plant chloroplasts, this quinone is part of the electron transfer chain in the light phase of photosynthesis and is capable of receiving two electrons. The analogs of SkQR1—SkQTR1 and SkQT1, containing toluquinone as an antioxidant head (Figure 2)—were also effective in reducing the neurological deficit caused by TBI in animals. It should be noted that C12TPP, which has a similar structure to SkQT1 but lacks the antioxidant group (Figure 2), did not exert a protective effect upon intraperitoneal administration to animals after TBI [30]. This experiment showed that the protective effects of SkQR1, SkQTR1, and SkQT1 are mediated by the antioxidant part of the molecule and not by its transport part, a penetrating cation associated with the linker.

More recently, it was demonstrated that even a single intravenous injection of SkQR1 after TBI improved the motor function of the limbs and increased survivability of neurons in the marginal layer of the lesion [26].

The effectiveness of using mitochondria-targeted antioxidants to treat TBI was confirmed by studies that used controlled cortical impact as a model of TBI followed by treatment with another brain-permeable mitochondria-targeted free radical scavenger, XJB-5-131 (known to prevent cardiolipin oxidation in the brain, as well as neuronal death both in vitro and in vivo), which markedly reduced behavioral deficits and cortical lesion volume [25]. In this work, the protector was administered 10 min after TBI at a dose of 3–25 µmol/kg, which is much higher than the effective concentrations of antioxidants of the SkQ group. As mentioned above, the cerebral circulatory system is very sensitive to damages that occur during TBI. It was shown that 24 h after TBI, the middle cerebral arteries exhibited impaired myogenic constriction, which was restored by treatment with the mitochondria-targeted antioxidant mitoTEMPO [31]. Recently, the data obtained from interesting experiments performed on the modified Marmarou weight-drop model of TBI, using the mitochondria-targeted antioxidant peptide SS-31 (5 mg/kg), were published [32]. Administration of this peptide 30 min after mild TBI significantly reversed mitochondrial dysfunction and ameliorated secondary brain injury caused by TBI. In the brain tissue, SS-31 directly decreased the ROS content, the level of malondialdehyde, and the release of cytochrome c and prevented the decline in the activity of superoxide dismutase, thus attenuating neurological deficits, brain water content, DNA damage, and neural apoptosis [32]. Using another mitochondria-targeted antioxidant (MitoQ, 6 µmol/kg), containing the active part of the CoQ10 molecule, the same authors showed that the neuroprotective effects of the mitochondria-targeted antioxidant in a model of mild closed TBI may be associated with the activation of NF-E2-related factor 2 (Nrf2)-antioxidant response element (ARE) pathway [33]. Nrf2 is involved in the activation of a group of antioxidant and detoxifying enzymes and genes that protect the body from the negative effects of oxidative stress. By binding to ARE, Nrf2 stimulates the expression of a variety of genes coding for phase II detoxifying and antioxidant enzymes, such as superoxide dismutase, heme oxygenase-1, glutathione peroxidase, and quinine oxidoreductase 1 [34,35,36,37]. Probably, the protective properties of mitochondria-targeted antioxidants are mediated by a number of protective mechanisms. For example, the protective effect of SkQR1 after its administration to animals is associated not only with the direct antioxidant effect of this substance, but also with its ability to stimulate the production of such a strong endogenous neuroprotector as erythropoietin (EPO), which leads to an increase in the phosphorylation of glycogen synthase kinase-3β in the brain, thus inactivating this proapoptotic enzyme [38]. It should be noted that the Nrf2 pathway may be the key mechanism mediating the protective effects of EPO [39]. Animals treated with EPO after TBI showed a significant reduction in the infiltration and activation of immune/inflammatory cells (neutrophils, CD3+T-cells, and microglia) in the injured hemisphere. In addition, EPO treatment led to an increase in the expression of the anti-inflammatory cytokine IL-10, as well as a decrease in the expression of the proinflammatory cytokines IL-1β and TNF-α in the injured brain tissue [40]. Moreover, using a mouse model of carrageenan-induced acute inflammation in the subcutaneous air pouch, SkQ1 (analog of SkQR1) was shown to exert a strong anti-inflammatory effect that manifested in a decrease in the absolute number of inflammatory cells, mainly neutrophils, and in their relative number, along with an increase in macrophage and mast cell content in the inflammatory exudate. The concentration of the proinflammatory cytokine IL-6 in the exudate tended to decrease as well. C12TPP produced no significant effect on the inflammation process [41]. TBI is known to cause a potent systemic inflammatory response that may lead to systemic damage and dysfunction/damage of adjacent tissues/organs and may even further exacerbate the secondary local damage [10,42,43]. Thus, the protective effect of SkQ in TBI may be associated with the anti-inflammatory properties of this substance. This conclusion is confirmed by the data obtained from the model of focal TBI in rats, which demonstrated that a single intravenous injection of SkQR1 after TBI prevented the increase in astroglial expression and reduced the infiltration of segmented neutrophils in the marginal layer of the lesion [26].

## 3. Conclusions

The data presented in these works demonstrate that mitochondria-targeted antioxidants are an effective means to reduce oxidative damages and the negative effects associated with the development of secondary damage caused by traumatic brain injury. Apparently, their protective action is mediated by both a rapid direct antioxidant effect and a slower stimulation of the endogenous defense systems of the body, such as an increase in the production of erythropoietin, the activation of the Nrf2-ARE pathway and, as a result, the inhibition of the inflammation process. In the future, not only a more detailed study of the mechanisms of action of various types of such antioxidants needs to be conducted, but also their therapeutic values and toxicological properties are to be determined. Moreover, the optimal therapeutic effect needs to be achieved in the shortest time possible from the onset of damage to the nervous tissue, since secondary brain damage in humans can develop for a long time, days and even months, depending on the severity of the damage.

## Abbreviations

TBI	Traumatic brain injury
ROS	Reactive oxygen species
Nrf2	NF-E2-related factor 2
ARE	Antioxidant response element
EPO	Erythropoietin
